# Extraction technique of trap states based on transient photo-voltage measurement

**DOI:** 10.1038/s41598-020-69914-y

**Published:** 2020-07-30

**Authors:** Zedong Lin

**Affiliations:** Department of Chemistry, Renmin University of China, Beijing, 100872 China

**Keywords:** Chemical physics, Electronic properties and materials

## Abstract

This article puts forward a technique for extracting the density of trap states (DOS_T_) distribution based on the transient photo-voltage (TPV) measurement result. We prove that when the TPV result is linear, the DOS_T_ distribution is exponential type and vice versa. Compared to the approach based on the space charge limited current measurement, the method given in this paper has the advantage of requiring less calculation. The results obtained by our method provides a guidance for preparing less trap states solar cells.

## Introduction

In order to improve the photo-voltaic performance of perovskite solar cells (PSCs), we need to further explore the mechanism such as carrier mobility^1–3^, ion migration^4–8^, density of trap states (DOS_T_) distribution^9–13^, carrier recombination^14–18^ and so on. The DOS_T_ distribution is a crucial factor that determines the photovoltaic performance of PSCs^19–33^. According to the literatures in recent years^19–23^, current density–voltage (*J–V*) hysteresis is caused by the ion migration and trap assisted carrier recombination. The DOS_T_ distribution affects the carrier recombination^24–27^, influences the open circuit voltage^28–30^, and hinders the enhancement of power conversion efficiency (PCE)^31–34^. The DOS_T_ distribution cannot be obtained by experimental measurement directly. There are only few methods for the extraction of DOS_T_ distribution. The space charge limited current (SCLC) method uses the deconvolution to extract the DOS_T_ distribution^35–40^, based on the measured *J*–*V* data at different temperatures. Walter et al.^41–45^ put forward the impedance spectroscopy (IS) method to extract the DOS_T_ distribution. They extract the DOS_T_ distribution based on the plot of equivalent chemical capacitance versus frequency given by IS measurement^41–45^. They regard the capture and de-capture of carrier by the trap states in the PSCs as charging and discharging of the equivalent chemical capacitance^41^. They put forward the formula *DOS*_T_ (*E*_*ω*_) = (*V*_bi_/*eW*)(d*C*/d*ω*)(*ω*/*k*_B_) to extract the DOS_T_ distribution^41–45^. Here, *C* is the equivalent chemical capacitance. *ω* is the angular frequency of the ac signal. *V*_bi_ is the built-in electric voltage. *W* is the depletion width. *k*_B_ is the Boltzmann constant. *e* is the elementary charge. The corresponding energy level is calculated by the formula *E*_*ω*_ = *k*_B_*T*ln(*ω*_0_/*ω*), where *T* is the ambient temperature and *ω*_0_ is the attempt-to-escape frequency^41–45^. Wang et al.^46^ proposed a transient photo-voltage (TPV) method for DOS_T_ distribution extraction. Based on the hypothesis of exponential type DOS_T_ distribution^46–49^, multiple-trapping model^46,50–52^, and the zero-temperature approximation^46,52^, they find that when the DOS_T_ distribution is exponential type, the logarithm of carrier lifetime and the photo-voltage satisfy linear relation. They use this relation to extract the DOS_T_ distribution based on the TPV result^46–48^. Because of the hypothesis of exponential type distribution^46–49^, their method is effective only when the TPV result is linear. The DOS_T_ distribution extracted by their method is an exponential type distribution^46–48^.

However, according to the TPV experiments reported in recent years^46–48^, the majority of TPV results are non-linear. In these cases, the method of Wang et al. is not effective to extract the DOS_T_ distribution. In this article, we put forward a new technique for extraction of DOS_T_ distribution based on the TPV measurement result. We give up the hypothesis of exponential type DOS_T_ distribution^46–49^ and zero-temperature approximation^46,52^. The method given in our work is based on the single hypothesis of multiple-trapping model^46,50–52^. Our method is effective for arbitrary TPV results and can be used to extract general type DOS_T_ distribution.

## Results and discussion

### Establishment of theory and method

In this section, we establish the equation for the extraction of DOS_T_ distribution based on the TPV result.

Because of the trap states in the perovskite absorber layer, the behavior of trapping and de-trapping of carrier by the trap states determines the recombination rate, which can be described by the multiple-trapping model^46,50–52^. According to the multiple-trapping model, the relation of carrier lifetime *τ*_n_ and the free carrier lifetime *τ*_f_ satisfies^46,50–52^

1 $$\tau _{{\text{n}}} = (\partial n/\partial n_{\text{c}} )\tau _{{\text{f}}} .$$

Here *n* = *n*_t_ + *n*_c_ denotes the sum of electron density in trap states *n*_t_ and electron density in conduct band *n*_c_.

Therefore, we have

2 $$\tau_{\text {n}} = (1 + \partial n_{\text{t}} /\partial n_{\text{c}} )\tau_{\text{f}} ,$$

which can be rewritten as

3 $$\tau_{{\text{n}}} = \left( {1 + \frac{{\partial n_{{\text{t}}} /\partial E_{{{\text{Fn}}}} }}{{\partial n_{{\text{c}}} /\partial E_{{{\text{Fn}}}} }}} \right)\tau_{{\text{f}}} .$$

The density of electron in trap states satisfies^46,52^

4 $$n_{{\text{t}}} = \int \rho_{{\text{t}}} \left( E \right)f\left( E \right)dE.$$

Here $\rho_{{\text{t}}} \left( E \right)$ denotes the DOS_T_ distribution. $f\left( E \right) = \frac{1}{{exp\left( {\frac{{E - E_{{{\text{Fn}}}} }}{{k_{B} T}}} \right) + 1}}$ denotes the Fermi–Dirac distribution^53^.

Therefore, we have

5 $$\frac{{\partial n_{{\text{t}}} }}{{\partial \left( {\frac{{E_{{{\text{Fn}}}} }}{{k_{{\text{B}}} T}}} \right)}} = \frac{\partial }{{\partial \left( {\frac{{E_{{{\text{Fn}}}} }}{{k_{{\text{B}}} T}}} \right)}}\left( {\int \rho_{{\text{t}}} \left( E \right)f\left( E \right)dE} \right).$$

According to the Fermi–Dirac distribution^53^, we have

6 $$\frac{{\partial n_{{\text{t}}} }}{{\partial \left( {\frac{{E_{{{\text{Fn}}}} }}{{k_{{\text{B}}} T}}} \right)}} = \int \rho_{{\text{t}}} \left( E \right)f\left( E \right)\left( {1 - f\left( E \right)} \right)dE.$$

We rewrite Eq. (6) as

7 $$\frac{{\partial n_{t} }}{{\partial E_{Fn} }} = \frac{1}{{k_{B} T}}\int \rho_{{\text{t}}} \left( E \right)f\left( E \right)\left( {1 - f\left( E \right)} \right)dE.$$

The carrier density in conductor band satisfies^46,52,53^

8 $$n_{{\text{c}}} = N_{{\text{c}}} exp\left( {\frac{{E_{{{\text{Fn}}}} - E_{{\text{c}}} }}{{k_{{\text{B}}} T}}} \right).$$

Here *N*_c_ is the density of effective states in conduction band. *E*_c_ is the conduction band energy level position^53^.

Therefore, we have

9 $$\partial n_{\text{c}} /\partial E_{\text{Fn}} = n_{\text{c}} /k_{\text{B}} T.$$

According to Eqs. (3), (7), and (9), we have

10 $$\tau_{{\text{n}}} = \left( {1 + \frac{{\int \rho_{{\text{t}}} \left( E \right)f\left( E \right)\left( {1 - f\left( E \right)} \right)dE}}{{n_{{\text{c}}} }}} \right)\tau_{{\text{f}}} .$$

We rewrite Eq. (10) as

11 $$\int \rho_{{\text{t}}} \left( E \right)f\left( E \right)\left( {1 - f\left( E \right)} \right)dE = \left( {\frac{{\tau_{{\text{n}}} }}{{\tau_{{\text{f}}} }} - 1} \right)n_{{\text{c}}} .$$

Substituting the Fermi–Dirac distribution into Eq. (11), we have

12 $$\int \rho_{{\text{t}}} \left( E \right)\frac{1}{{exp\left( {\frac{{E - E_{{{\text{Fn}}}} }}{{k_{B} T}}} \right) + 1}}\left( {1 - \frac{1}{{exp\left( {\frac{{E - E_{{{\text{Fn}}}} }}{{k_{B} T}}} \right) + 1}}} \right)dE = \left( {\frac{{\tau_{{\text{n}}} }}{{\tau_{{\text{f}}} }} - 1} \right)n_{{\text{c}}} .$$

We rewrite Eq. (12) as

13 $$\int \rho_{{\text{t}}} \left( E \right)\frac{1}{{exp\left( {\frac{{ - \left( {E_{{{\text{Fn}}}} - E} \right)}}{{k_{B} T}}} \right) + 1}}\left( {1 - \frac{1}{{exp\left( {\frac{{ - \left( {E_{{{\text{Fn}}}} - E} \right)}}{{k_{B} T}}} \right) + 1}}} \right)dE = \left( {\frac{{\tau_{{\text{n}}} }}{{\tau_{{\text{f}}} }} - 1} \right)n_{{\text{c}}} .$$

We define a derivation factor

14 $$g\left( {E_{{{\text{Fn}}}} - E} \right) = \frac{1}{{exp\left( {\frac{{ - \left( {E_{{{\text{Fn}}}} - E} \right)}}{{k_{B} T}}} \right) + 1}}\left( {1 - \frac{1}{{exp\left( {\frac{{ - \left( {E_{{{\text{Fn}}}} - E} \right)}}{{k_{B} T}}} \right) + 1}}} \right).$$

and rewrite Eq. (13) as

15 $$\int \rho_{{\text{t}}} \left( E \right)g\left( {E_{{{\text{Fn}}}} - E} \right)dE = \left( {\frac{{\tau_{{\text{n}}} }}{{\tau_{{\text{f}}} }} - 1} \right)n_{{\text{c}}} .$$

According to the TPV result, the carrier lifetime is a function of photo-voltage^46–49^. Therefore, we rewrite Eq. (15) as

16 $$\int \rho_{{\text{t}}} \left( E \right)g\left( {E_{{{\text{Fn}}}} - E} \right)dE = \left( {\frac{{\tau_{{\text{n}}} \left( {V_{{{\text{ph}}}} } \right)}}{{\tau_{{\text{f}}} }} - 1} \right)n_{{\text{c}}} .$$

Equation (16) is the fundamental equation of our method. The right-hand side of Eq. (16) can be obtained from experimental measurement. *τ*_n_(*V*_ph_) can be measured from TPV experiment. *n*_c_ can be obtained from differential charging method^5^ or from SCLC under different intensity of illumination^39^. We can use absorbance spectrum, Kelvin probe (KP), ultraviolet photoemission spectroscopy (UPS), and X-ray photoelectron spectroscopy (XPS) to get conduction band energy level position *E*_c_, valence band energy level position *E*_v_, Fermi energy level position *E*_F0_ and band gap *E*_g_^35^, respectively. After getting the conductor band electron density in dark *n*_0_ and in different intensity of illumination *n*_c_, according to the relation *E*_Fn_ = *E*_F0_ + *k*_B_*Tln*(*n*_c_/*n*_0_)^53^, we obtain the electron quasi Fermi energy level *E*_Fn_ corresponding to the photo-voltage *V*_ph_ in the different intensity of illumination. The free carrier lifetime *τ*_f_ can be measured or can be calculated according to the equation*τ*_f_ = 1/*C*_n_*N*_t_^54^. Here, *N*_t_ is the electron trap concentration. *C*_n_ denotes the capture coefficients for electrons. Since the left-hand side of Eq. (16) is the convolution integral of the DOS_T_ distribution and the derivative factor, we can get the DOS_T_ distribution by deconvolution. We use the numerical deconvolution function ([q,r] = deconv(u,v)) of MATLAB to solve the equation for DOS_T_ distribution.

For intrinsic perovskite, the electron density in conductor band satisfies^53^

17 $$n_{{\text{c}}} = N_{{\text{c}}} exp\left( {\frac{{E_{{{\text{Fn}}}} - E_{{{\text{cb}}}} }}{{k_{{\text{B}}} T}}} \right) = n_{0} exp\left( {\frac{{V_{{{\text{ph}}}} e}}{{2k_{{\text{B}}} T}}} \right).$$

Here $n_{0} = N_{{\text{c}}} exp\left( {\frac{{E_{{{\text{F}}0}} - E_{{{\text{cb}}}} }}{{k_{{\text{B}}} T}}} \right)$ is the electron density in conductor band at dark state^53^.

Substituting Eq. (17) into Eq. (16), we have

18 $$\int \rho_{{\text{t}}} \left( E \right)g\left( {E_{{{\text{Fn}}}} - E} \right)dE = \left( {\frac{{\tau_{{\text{n}}} \left( {V_{{{\text{ph}}}} } \right)}}{{\tau_{{\text{f}}} }} - 1} \right)n_{0} exp\left( {\frac{{V_{{{\text{ph}}}} e}}{{2k_{{\text{B}}} T}}} \right).$$

Equation (18) is the fundamental equation for DOS_T_ distribution extraction of intrinsic perovskite. Similarly, we obtain the DOS_T_ distribution using numerical deconvolution function in MATLAB. The parameters used for calculation are list in Table 1^27,35^.

**Table 1:** Parameters used for DOS_T_ distribution calculation^27,35^.

Sym	Description	Value
*e*	Elementary charge	1.60 × 10^–19^ C
*k*_B_	Boltzmann constant	1.38 × 10^–23^ JK^−1^
*E*_c_	Conduction band minimum	− 4.18 eV
*E*_g_	Band gap	1.52 eV
*E*_F0_	Fermi energy level	− 4.3 eV
* T*	Ambient temperature	300 K
*N*_c_	Conduction band DoS	1 × 10^24^ m^−3^
*C*_n_	Electron trap coefficients	1 × 10^–15 ^m^3 ^s^−1^
*N*_t_	Electron trap concentration	1 × 10^19^ m^−3^

### Exponential type DOS_T_ distribution

In this section, we explore the exponential type DOS_T_ distribution. Wang et al.^46^ find that for the exponential type DOS_T_ distribution, the logarithm of carrier lifetime is proportional to the photo-voltage (TPV result). The proof of this relation is shown as follows.

The exponential type DOS_T_ distribution satisfies^46–49^

19 $$\rho_{{\text{t}}} \left( E \right) = \frac{{N_{T} }}{{E_{{\text{B}}} }}exp\left( {\frac{{E - E_{{\text{c}}} }}{{E_{{\text{B}}} }}} \right).$$

Here, *E*_B_ is the characteristic energy and *N*_T_ is the total density of the trapped state. Substituting formula (19) into formula (4), and taking zero-temperature approximation^46,52^, we have

20 $$n_{{\text{T}}} = N_{{\text{T}}} \left( {exp\left( {\frac{{E_{{{\text{Fn}}}} - E_{{\text{c}}} }}{{E_{{\text{B}}} }}} \right) - exp\left( {\frac{{E_{{\text{v}}} - E_{{\text{c}}} }}{{E_{{\text{B}}} }}} \right)} \right).$$

Making the approximation of *n* ≈ *n*_T_^50^, we rewrite the multiple-trapping model^46,50–52^ as

21 $$\tau_{\text{n}} = (\partial n_{\text{T}} /\partial n_{\text{c}} )\tau_{\text{f}} ,$$

which is equivalent to

22 $$\tau_{{\text{n}}} = \frac{{\partial n_{{\text{T}}} /\partial V_{{{\text{ph}}}} }}{{\partial n_{{\text{c}}} /\partial V_{{{\text{ph}}}} }}\tau_{{\text{f}}} .$$

The electron density in conductor band satisfies^53^

23 $$n_{{\text{c}}} = N_{{\text{c}}} exp\left( {\frac{{E_{{{\text{Fn}}}} - E_{{\text{c}}} }}{{k_{{\text{B}}} T}}} \right).$$

According to Eqs. (20), (22), and (23) and from the relation of *E*_Fn_ = *E*_Fp_ + *eV*_ph_, we have

24 $$ln \tau_{\text{n}} = \left( {e/E_{\text{B}} - e/k_{\text{B}} T} \right)V_{\text{ph}} + lnA.$$

Here $$A = \frac{{N_{{\text{T}}} k_{{\text{B}}} T}}{{N_{{\text{c}}} E_{{\text{B}}} }}exp\left( {\frac{{E_{{{\text{Fp}}}} - E_{{\text{c}}} }}{{E_{{\text{B}}} }} - \frac{{E_{{{\text{Fp}}}} - E_{{\text{c}}} }}{{k_{{\text{B}}} T}}} \right)\tau_{{\text{f}}}$$.

We write Eq. (24) as linear mathematical form *lnτ*_n_ = *aV*_ph_ + *b*.

Here, *a* = *e*/*E*_B_ − *e*/*k*_B_*T*, *b* = *lnA*.

Therefore, we finish the proof of this relation. Note that for the intrinsic perovskite, Eq. (24) can be written as

25 $$ln \tau_{\text{n}} = \left( {e/2E_{\text{B}} - e/2k_{\text{B}} T} \right)V_{\text{ph}} + lnA.$$

Here $A = \frac{{N_{{\text{T}}} k_{{\text{B}}} T}}{{N_{{\text{c}}} E_{{\text{B}}} }}exp\left( {\frac{{E_{{{\text{F}}0}} - E_{{\text{c}}} }}{{E_{{\text{B}}} }} - \frac{{E_{{{\text{F}}0}} - E_{{\text{c}}} }}{{k_{{\text{B}}} T}}} \right)\tau_{{\text{f}}}$. We write Eq. (25) as linear form *lnτ*_n_ = *aV*_ph_ + *b*. Here, *a* = *e*/2 *E*_B_− *e*/2*k*_B_*T*, *b* = *lnA*.

Similarly, we can also prove that when TPV result is linear, the extracted DOS_T_ distribution is exponential type. Details of the proof are given in the supporting information. We can use this relation to extract the DOS_T_ distribution when the TPV result is linear. Below, this method is called analytic method.

**Figure 1:**
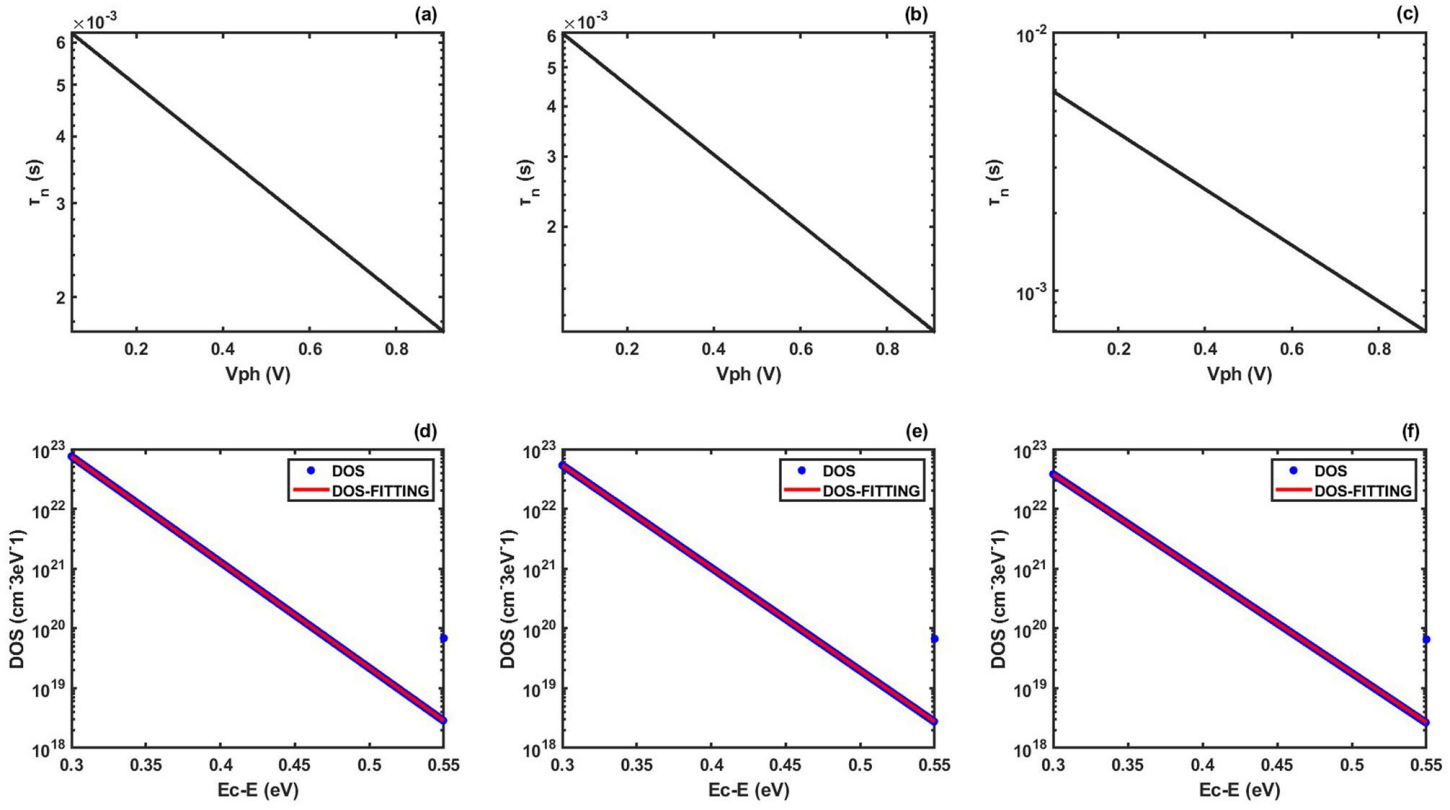
(**a**)–(**c**) Plots of carrier lifetime versus photo-voltage. We set *b* =  − 5 and *a* as − 1.5, − 2 and − 2.5, respectively. (**d**)–(**f**) DOS_T_ distributions extracted from (**a**)–(**c**), respectively. The blue dots represent the extracted DOS_T_ distributions, the red lines represent the exponential fitting of extracted DOS_T_ distributions.

We can use the analytic method to verify the validity of our numerical method. We use both our numerical method and the analytic method to extract the DOS_T_ distribution and make a comparison. In Fig. 1a–c, we set the *b* = − 5 and the slope parameter *a* as − 1.5, − 2 and − 2.5, respectively. Using the analytic method, we derive *E*_B_ and *N*_T_ (see Table 2). Figure 1d–f shows the extracted DOS_T_ distributions from Fig. 1a–c using our numerical method. As expected by the analytic method, the DOS_T_ distribution is exponential type. In order to compare with the DOS_T_ distribution extracted by analytic method, we use the exponential fitting (*f*(*E*) = *cexp*(*dE*)) to calculate the *E*_B_ and *N*_T_ (see Table 2). As shown in Table 2, the *E*_B_ and *N*_T_ calculated by our numerical method are consistent with the *E*_B_ and *N*_T_ calculated by the analytic method, indicating that the numerical algorithm to do the deconvolution in our calculation is reliable. We explain the slight deviation of *E*_B_ and *N*_T_ extracted by the two methods as follows. The analytic method is established based on the three hypothesis of multiple-trapping model^46,50–52^, zero-temperature approximation^46,52^, and exponential type DOS_T_ distribution^46–49^. Our numerical method is established based on the unique hypothesis of multiple-trapping model^46,50–52^. Therefore, the slight deviation of *E*_B_ and *N*_T_ extracted by the two methods is attributed to the zero-temperature approximation and the fitting error.

**Table 2:** Exponential fitting coefficients, distribution coefficients calculated from Fig. 2.

Coefficients	Example 1	Example 2	Example 3
**Chosen parameters**			
*a*	− 1.5	− 2.0	− 2.5
*b*	− 5	− 5	− 5
**Exponential fitting**			
*c* (exponential fitting)	1.525 × 10^34^	7.536 × 10^33^	3.666 × 10^33^
*d* (exponential fitting)	− 2.542 × 10^20^	− 2.466 × 10^20^	− 2.388 × 10^20^
R-square	1	1	1
**Calculation result**			
*E*_B_(analytic method)	28.0 meV	28.8 meV	29.7 meV
*E*_B_(our method)	24.6 meV	25.3 meV	26.1 meV
*N*_T_(analytic method)	7.3050 × 10^13^ cm^−3^	7.5158 × 10^13^ cm^−3^	7.7392 × 10^13^ cm^−3^
*N*_T_(our method)	5.9992 × 10^13^ cm^−3^	3.0560 × 10^13^ cm^−3^	1.5352 × 10^13^ cm^−3^

### Non-exponential type DOS_T_ distribution

**Figure 2:**
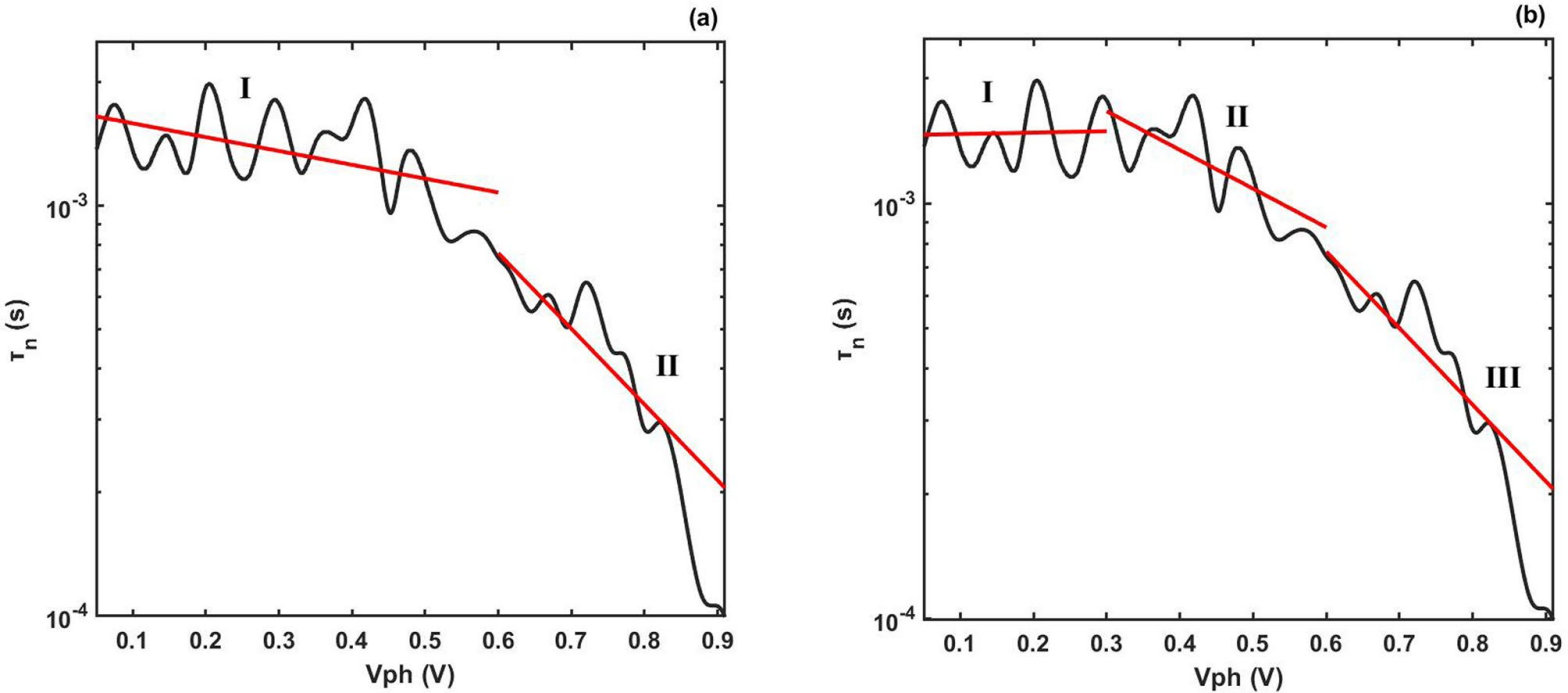
Plots of carrier lifetime versus photo-voltage given by TPV measurement^47^. The black lines represent the result of TPV measurement. The red lines represent linear fitting in the subintervals. (**a**) shows the linear fittings of TPV in two subintervals, respectively. (**b**) shows the linear fittings of TPV in three subintervals, respectively.

In this section, we investigate the non-exponential type DOS_T_ distribution. We take the TPV data in Ref.^47^ as an example. As shown in Fig. 2a,b, the TPV result is non-linear. Hence, we cannot use the analytic method to extract the DOS_T_ distribution.

To overcome this difficulty, Wang et al.^47^ made a linear fitting in the subintervals of 0.05–0.6 V and 0.6–0.91 V (see Fig. 2a). They used formula (24) to get two values of *E*_B_ in these two subintervals, respectively^47^. They explained these two *E*_B_ as two types of exponential type DOS_T_ distribution (They called them as deep trap type and shallow trap type)^47^. However, there are some difficulties in their method. (1) Equation (24) is derived from one exponential type DOS_T_ distribution, not the two types of DOS_T_ distribution (deep trap state type DOS_T_ distribution and shallow trap state type DOS_T_ distribution). We cannot derive Eq. (24) based on these two types of distribution. (2) There is no clear boundary for deep and shallow trap. So the definition of deep and shallow trap is ambiguous. (3) It is more accurate to take linear fittings in three subintervals, respectively (see Fig. 2b). According to the differential theory, we can divide the photo-voltage interval into infinite differential subintervals and use Eq. (24) to calculate the *E*_B_ in these differential subintervals, respectively. These *E*_B_ cannot be explained by the concept of deep and shallow trap.

**Figure 3:**
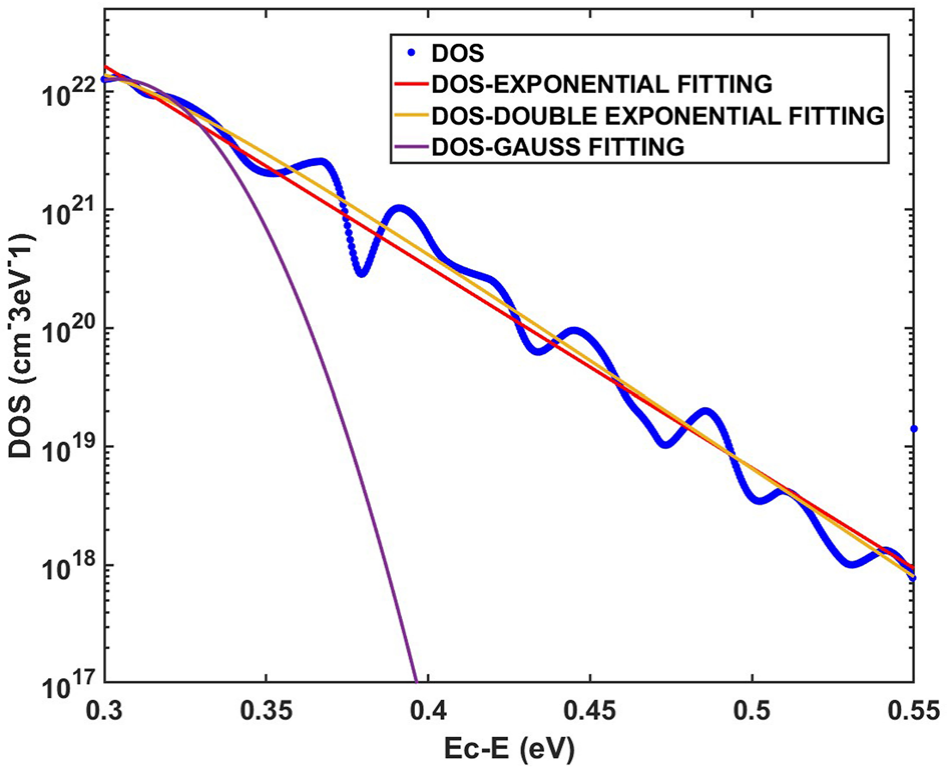
DOS_T_ distribution calculated by our method given in this article. The blue dots represent the extracted DOS_T_ distribution. The red, yellow and purple lines represent the exponential, double exponential and Gauss fittings for the extracted DOS_T_ distribution, respectively.

Our method is effective for arbitrary TPV results, which can be used to extract general type DOS_T_ distribution. Using our method, we extract the DOS_T_ distribution (as illustrated in Fig. 3). It can be seen that the extracted DOS_T_ distribution is not an exponential type distribution. Compared to the method given by Wang et al., our method is more accurate and it can give the fine structure of DOS_T_ distribution.

We make the exponential, double exponential and Gauss fittings for the extracted DOS_T_ distribution, respectively (see Fig. 3). The R-square (Coefficient of determination) of these fittings are given in Table 3. It can be see that the R-square of double exponential fitting is larger than the exponential fitting and Gauss fitting, indicating that the DOS_T_ distribution is more consistent with the double exponential type distribution than with the exponential type distribution. Therefore, when the TPV result is non-linear, the extracted DOS_T_ distribution is non-exponential type.

**Table 3:** Different type fitting for extracted DOS_T_ distribution.

Fitting type	Exponential	Double exponential	Gauss
R-square	0.9679	0.9864	0.9331

### Calculation example

**Figure 4:**
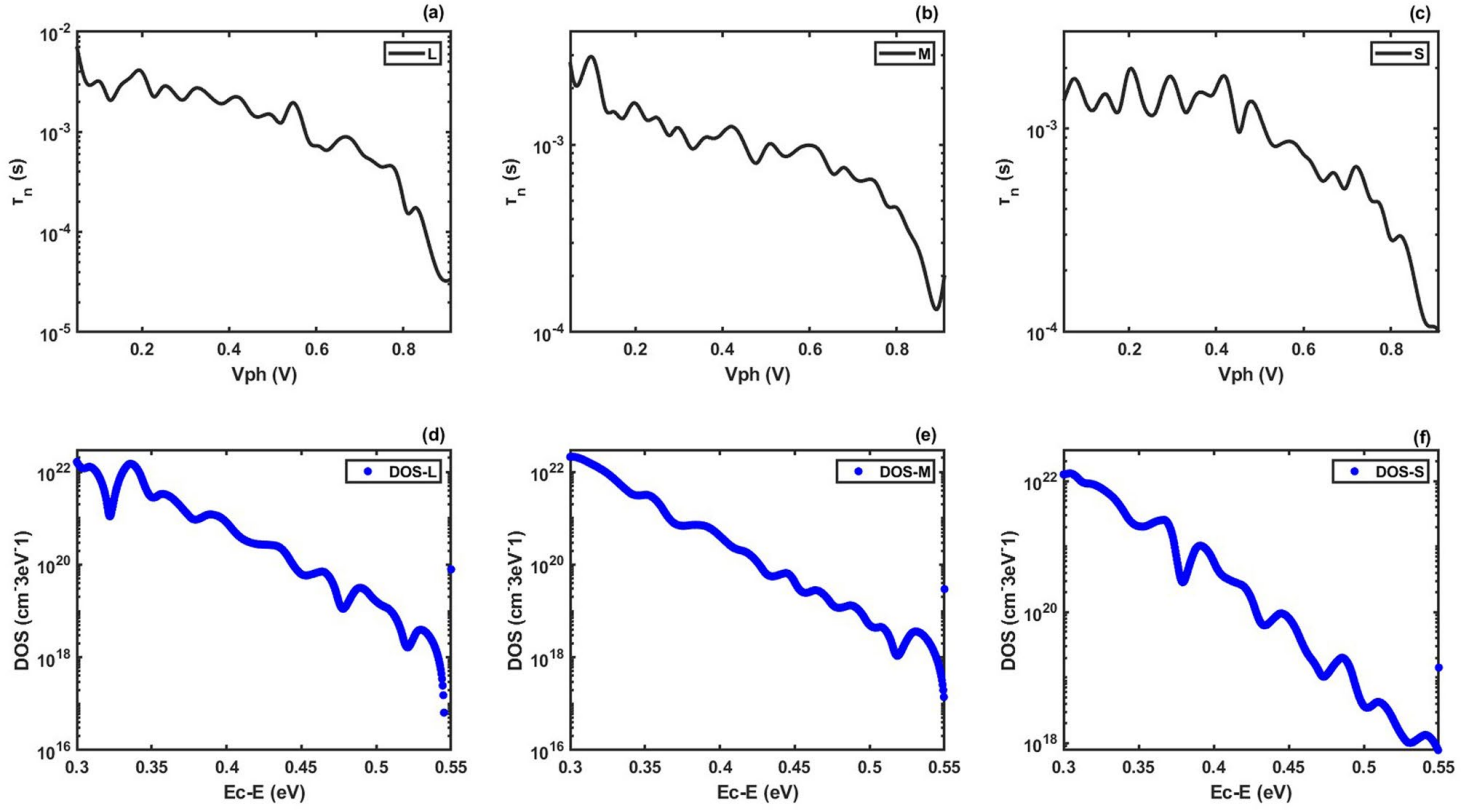
(**a**)–(**c**) TPV results for meso-structured perovskite solar cells with large, middle and small size of perovskite grain^47^. (**d**)–(**f**) DOS_T_ distributions extracted by our method from (**a**)–(**c**), respectively.

In this section, we give some examples of DOS_T_ distribution extraction using our method. We take the TPV data in Ref.^47^ as an example. Figure 4a–c shows the TPV results for meso-structured perovskite solar cells with large, middle and small size of perovskite grain, respectively. Using our method, we extract the DOS_T_ distributions from Fig. 4a–c, respectively, (as shown in Fig. 4d–f).

**Figure 5:**
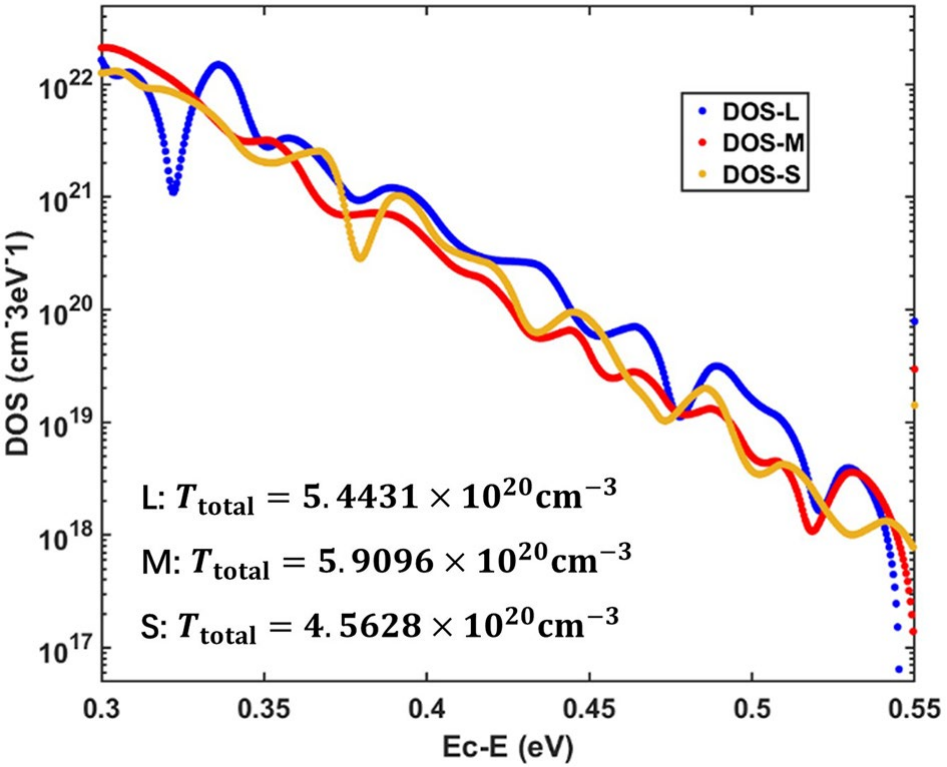
DOS_T_ distributions for meso-structured perovskite solar cells with large, middle and small size of perovskite grain extracted by our method given in this paper.

For comparing, we plot the extracted DOS_T_ distributions in Fig. 5. Using the formula ${T}_{\mathrm{total}}=\int {\rho }_{\mathrm{t}}\left(E\right)dE$, we calculate the total amount of trap states for three solar cells. We derived *T*_total_ = 5.4431 × 10^20^ cm^−3^ (large size), *T*_total_ = 5.9096 × 10^20^ cm^−3^ (middle size) and *T*_total_ = 4.5628 × 10^20^ cm^−3^ (small size). It can be seen that the solar cell with small size of perovskite grain has the least amount of trap states. This result could give a guidance for preparing perovskite solar cells with less trap states.

### Comparison to SCLC method

SCLC measurement gives the relation of current density and voltage at different temperatures (*j* = *j* (*U*, *T*)). For SCLC method, we extract the DOS_T_ distribution adopting the equations listed as follows^35–40^

26 $$E_{\text{a}} = - dlnj/d\left( {k_{\text{B}} T} \right)^{ - 1} ,$$

27 $$m = d\left( {lnj} \right)/d\left( {lnU} \right),$$

28 $$B = - \left[ {dm/d\left( {lnU} \right)} \right]/\left[ {m\left( {m - 1} \right)\left( {2m - 1} \right)} \right],$$

29 $$C = \left( {B\left( {2m - 1} \right) + B^{2} \left( {3m - 2} \right) + d\left( {ln\left( {1 + B} \right)} \right)/d\left( {lnU} \right)} \right)/\left( {1 + B\left( {m - 1} \right)} \right),$$

30 $$\int \rho_{{\text{t}}} \left( E \right)f\left( E \right)\left( {1 - f\left( E \right)} \right)dE = \frac{1}{{k_{{\text{B}}} T}}\frac{\varepsilon U}{{eL^{2} }}\frac{2m - 1}{{m^{2} }}\left( {1 + C} \right).$$

Here *j* denotes the current density. *U* denotes the voltage. *E*_a_ is the activation energy. *m*, *B*, *C* are the parameters for calculation. *L* is the thickness of perovskite absorber layer. *e* is the elementary charge. *ε* is the dielectric constant of perovskite absorber layer. Based on the SCLC measurement result, we need to calculate *E*_a_, *m*, *B*, *C* and finally use Eq. (30) to extract the DOS_T_ by deconvolution^35–40^. The calculations of *E*_a_, *m*, *B*, *C* are complicated. For our method, we only need to use formula (18) to extract the DOS_T_ distribution by deconvolution. Therefore, our method takes less computation.

## Conclusion

In conclusion, this article presents a new technique for DOS_T_ distribution extraction based on the TPV measurement result. The approach given in this paper is effective for extraction of general type DOS_T_ distribution. We prove that when the TPV result is linear, the DOS_T_ distribution is exponential type and vice versa. Our method needs less computation than the SCLC method. The obtained results provide a guidance for preparing perovskite solar cells with less trap states.

## Methods

**Figure 6:**
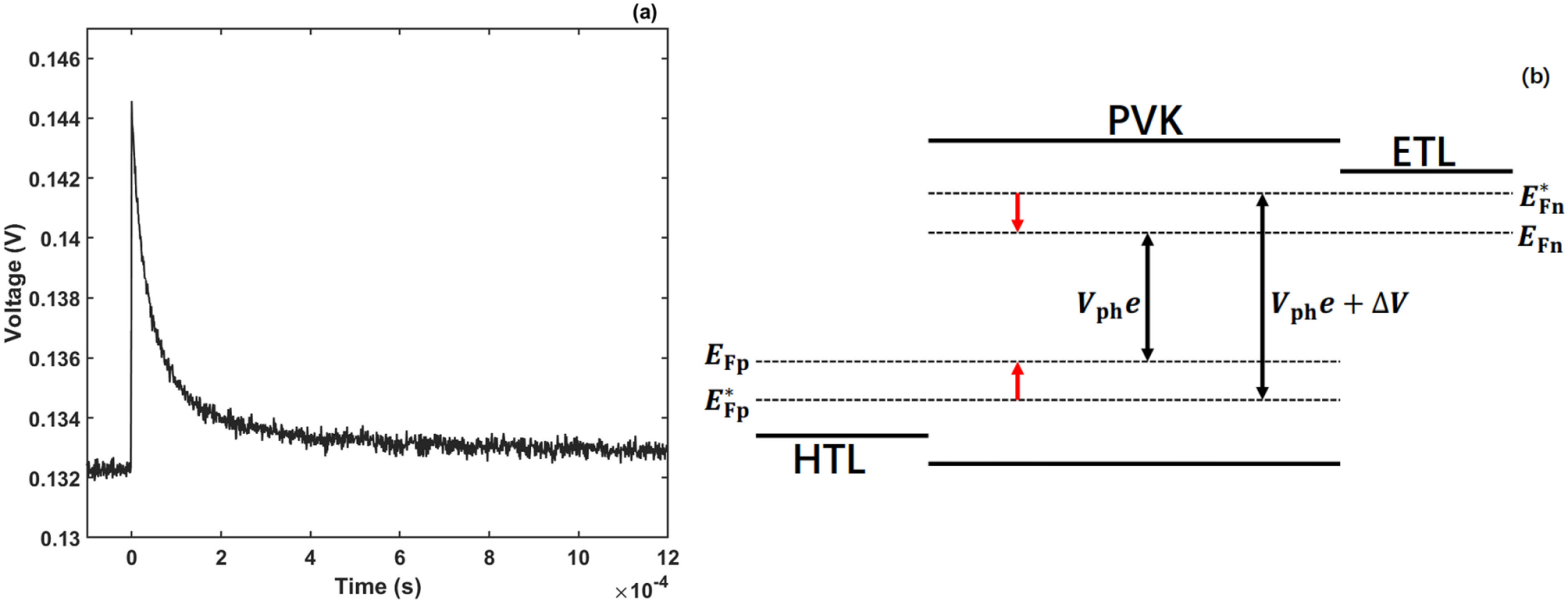
(**a**) Result of TPV measurement. (**b**) Mechanism of TPV experiment.

The transient photo-voltage measurement is an effective technique for the study of carrier recombination^3,46–49^. Figure 6a shows the typical TPV measurement result. Figure 6b shows the mechanism of TPV experiment. Photo-voltaic device is held under open circuit. At the start of the TPV experiment, we use a steady-state bias light to illuminate the device until the equilibrium between generation and recombination is established. The steady-state bias light produces a bias photo-voltage *V*_ph_. As a result, the Fermi energy level *E*_F0_ changes to electron quasi Fermi energy level *E*_Fn_ and hole quasi Fermi energy level *E*_Fp_. Thereafter, we apply an additional small light pulse to the device. With the perturbation of small light pulse, the photo-voltage increases to *V*_ph_ + Δ*V*. The electron quasi Fermi energy level *E*_Fn_ shifts to ${E}_{\mathrm{Fn}}^{*}$, and the hole quasi Fermi energy level *E*_Fp_ to ${E}_{\mathrm{Fp}}^{*}$. After switching off the small light pulse source, due to carrier recombination, the photo-voltage decays exponentially until it reaches the bias value *V*_ph_. The electron quasi Fermi energy level comes back to *E*_Fn_ from ${E}_{\mathrm{Fn}}^{*}$ and the hole quasi Fermi energy level comes back to *E*_Fp_ from ${E}_{\mathrm{Fp}}^{*}$.The carrier lifetime *τ*_n_ is defined as the time taken for the photo-voltage to decay from *V*_ph_ + Δ*V* to *V*_ph_ + Δ*V*/e. Here, e is the natural constant. By tuning the intensity of steady-state bias light *I*, we get the relation between the photo-voltage *V*_ph_ and carrier lifetime *τ*_n_, which is written as *τ*_n_ = *τ*_n_(*V*_ph_) (TPV result)^46–49^.

## Supplementary information

Supplementary Information.
