# Supplementary material for: Extraction technique of trap states based on transient photo-voltage measurement
Source: Sci Rep. 2020 Jul 30;10:12888. doi: 10.1038/s41598-020-69914-y (PMC7393153; doi:10.1038/s41598-020-69914-y)
Supplement: Supplementary file 1 — Supplementary Information. [file 41598_2020_69914_MOESM1_ESM.docx]

**Supporting Information**

**Extraction Technique of Trap States Based on Transient Photo-Voltage Measurement**

Zedong Lin

Department of Chemistry, Renmin University of China, Beijing 100872, China.

E-mail: zedonglin@ruc.edu.cn

In this document, we prove the relation that when the TPV result is linear, the extracted DOS_T_ distribution is exponential type.

The linear TPV result satisfies

*lnτ*_n_=(*e*/*E*_B_-*e*/*k*_B_*T*)*V*_ph_+*lnA*. (S1)

Here, $A=\frac{N_{T}k_{B}T}{N_{c}E_{B}}exp\left( \frac{E_{\mathrm{Fp}}-E_{c}}{E_{B}}-\frac{E_{\mathrm{Fp}}-E_{c}}{k_{B}T} \right)\tau_{f}$.

We rewrite equation (S1) as

$\tau_{n}=\frac{N_{T}T}{N_{c}T_{c}}exp\left( \frac{E_{\mathrm{Fn}}-E_{c}}{k_{B}T_{c}}-\frac{E_{\mathrm{Fn}}-E_{c}}{k_{B}T} \right)\tau_{f}$. (S2)

By taking the approximation of *n*≈*n*_T_^1^, we rewrite the multiple-trapping model^1-4^ as

$\tau_{n}=\frac{\partial n_{T}}{\partial n_{c}}\tau_{f}$. (S3)

We rewrite equation (S3) as

$\tau_{n}=\frac{{\partial n_{T}}/{\partial V_{\mathrm{ph}}}}{{\partial n_{c}}/\partial V_{\mathrm{ph}}}\tau_{f}$. (S4)

According to formula (S2) and (S4), we have

$\frac{{\partial n_{T}}/{\partial V_{\mathrm{ph}}}}{{\partial n_{c}}/\partial V_{\mathrm{ph}}}=\frac{N_{T}T}{N_{c}T_{c}}exp\left( \frac{E_{\mathrm{Fn}}-E_{c}}{k_{B}T_{c}}-\frac{E_{\mathrm{Fn}}-E_{c}}{k_{B}T} \right)$. (S5)

Using $n_{c}=N_{c}exp\left( \frac{E_{\mathrm{Fn}}-E_{c}}{k_{B}T} \right)$^5^, we have

$\frac{\partial n_{c}}{\partial V_{\mathrm{ph}}}=\frac{N_{c}e}{k_{B}T}exp\left( \frac{E_{\mathrm{Fn}}-E_{c}}{k_{B}T} \right)$. (S6)

Substituting formula (S6) into formula (S5), we have

$\frac{\partial n_{T}}{\partial V_{\mathrm{ph}}}=\frac{N_{T}e}{k_{B}T_{c}}exp\left( \frac{E_{\mathrm{Fn}}-E_{c}}{k_{B}T_{c}} \right)$, (S7)

which is equivalent to

$\frac{\partial n_{T}}{\partial\left( eV_{\mathrm{ph}} \right)}=\frac{N_{T}}{k_{B}T_{c}}exp\left( \frac{E_{\mathrm{Fn}}-E_{c}}{k_{B}T_{c}} \right)$. (S8)

According to the relation of *E*_Fn_=*E*_Fp_+*eV*_ph_, we rewrite equation (S8) as

$\frac{\partial n_{T}}{\partial E_{\mathrm{Fn}}}=\frac{N_{T}}{k_{B}T_{c}}exp\left( \frac{E_{\mathrm{Fn}}-E_{c}}{k_{B}T_{c}} \right)$. (S9)

Therefore, we have

$\frac{dn_{t}}{dE}=\frac{N_{T}}{k_{B}T_{c}}exp\left( \frac{E-E_{c}}{k_{B}T_{c}} \right)$. (S10)

According to the definition of DOS_T_ distribution $\rho_{t}\left( E \right)={dn_{t}}/{dE}$, we obtain

$\rho_{t}\left( E \right)=\frac{N_{T}}{k_{B}T_{c}}exp\left( \frac{E-E_{c}}{k_{B}T_{c}} \right)$, (S11)

which completes the proof.

**References**

1. Bisquert, J. & Vikhrenko, V. S. Interpretation of the time constants measured by kinetic techniques in nanostructured semiconductor electrodes and dye-sensitized solar cells. *J. Phys. Chem. B* **108,** 2313-2322 (2004).
2. Wang, Y. *et al*. Correlation between energy and spatial distribution of intragap trap states in the tio2 photoanode of dye-sensitized solar cells. *Chem. Phys. Chem*. **16,** 2253-2259 (2015).
3. Bisquert, J., Fabregat-Santiago, F., Mora-Seró, I., Garcia-Belmonte, G. & [Giménez](https://pubs.acs.org/action/doSearch?field1=Contrib&text1=Sixto++Gim%C3%A9nez), S. Electron lifetime in dye-sensitized solar cells: theory and interpretation of measurements. *J. Phys. Chem. C* **113,** 17278-17290 (2009).
4. Wang, H. *et al*. Multiple-trapping model for the charge recombination dynamics in mesoporous-structured perovskite solar cells. *Chem. Sus. Chem*. **10,** 4872-4878 (2017).
5. Nelson, J. *The Physics of Solar Cells.* (Imperial College Press, 2003).
